# Primary Peritoneal Tuberculosis in an Immunocompetent Patient: A Case Report

**DOI:** 10.7759/cureus.101940

**Published:** 2026-01-20

**Authors:** Vijayakumaran Yanusha, Pakkiyaretnam Mayurathan

**Affiliations:** 1 Internal Medicine, Batticaloa Teaching Hospital, Batticaloa, LKA; 2 University Medical Unit, Batticaloa Teaching Hospital, Batticaloa, LKA; 3 Clinical Sciences, Faculty of Healthcare Sciences, Eastern University Sri Lanka, Batticaloa, LKA

**Keywords:** antituberculosis, biopsy, immunocompetent, paracentesis, tuberculosis

## Abstract

Tuberculosis (TB) is endemic in Sri Lanka but is associated with a comparatively low overall disease burden. Pulmonary TB is the most common manifestation among immunocompetent individuals, whereas peritoneal TB is rare and presents significant diagnostic challenges due to nonspecific clinical features such as abdominal distension, chronic diarrhoea, and ascites. We report the case of a 33-year-old immunocompetent male who presented with progressive, painless abdominal distension. A diagnostic mini-laparotomy with omental biopsy confirmed peritoneal TB. Anti-tuberculous therapy was initiated, resulting in complete resolution of ascites on follow-up. This case highlights the diagnostic difficulty of abdominal TB in immunocompetent patients and emphasizes the importance of maintaining a heightened suspicion and initiating early treatment to improve clinical outcomes.

## Introduction

Tuberculosis (TB) continues to be a major public health problem worldwide, particularly in developing countries such as Sri Lanka. The disease is endemic in Sri Lanka, with recent epidemiological studies estimating an incidence of 63 cases per 100,000 population, corresponding to approximately 14,000 new TB cases [1]. Nearly one-fourth of the global population (i.e., approximately two billion people) is estimated to be infected with *Mycobacterium tuberculosis* and is at risk of developing the disease [2]. It is an infectious disease caused by the bacillus *Mycobacterium tuberculosis* (MTB) and occasionally by *Mycobacterium bovis* and *Mycobacterium africanum* [3]. Most cases of TB present as pulmonary TB. Abdominal TB is a form of extrapulmonary TB that involves the GI tract, peritoneum, intra-abdominal lymph nodes, and visceral organs, either singly or in combination [3]. Timely diagnosis and prompt initiation of standard anti-tuberculous therapy are crucial to prevent complications such as intestinal obstruction, fistula formation, and bowel perforation [3].

## Case presentation

A 33-year-old previously healthy male with no history of illicit drug use presented with progressive, painless abdominal distension for one month. On examination, he was febrile, with gross ascites and stony dullness over the left lower lung zone accompanied by reduced breath sounds. Examination of other systems was unremarkable. Laboratory tests showed a normal full blood count except for mild anemia, and biochemical tests were within normal limits except for hypoalbuminemia (albumin 18 g/L), elevated CRP (76 mg/L), and ESR (55 mm/hr). An abdominal ultrasound revealed mild to moderate septated free fluid in the epigastrium and pelvis, with no organomegaly or deposits. Viral serologies for HBsAg, anti-HBs, anti-HCV, anti-HIV, anti-HAV IgM, and retroviral screening were all negative. Diagnostic paracentesis showed a lymphocyte-predominant exudative ascitic fluid with no bacterial growth (Table 1).

**Table 1:** Ascitic fluid analysis. Ascitic fluid laboratory parameters obtained at the time of admission. The analysis demonstrates elevated protein content and lymphocyte-predominant leukocytosis, findings suggestive of an exudative ascitic fluid. Glucose levels were within the expected range, and red cell counts were not significantly elevated.

Ascitic fluid contents	On admission value	Reference range
Protein	52 g/L	<25 g/L
Glucose	5.4 mmol/L	3.3-5.6 mmol/L
Red cells	2,500/mm³	<10,000/mm³
White cells	740/mm³ (15 polymorphs, 725 lymphocytes)	<250/mm³

**Figure 1:**
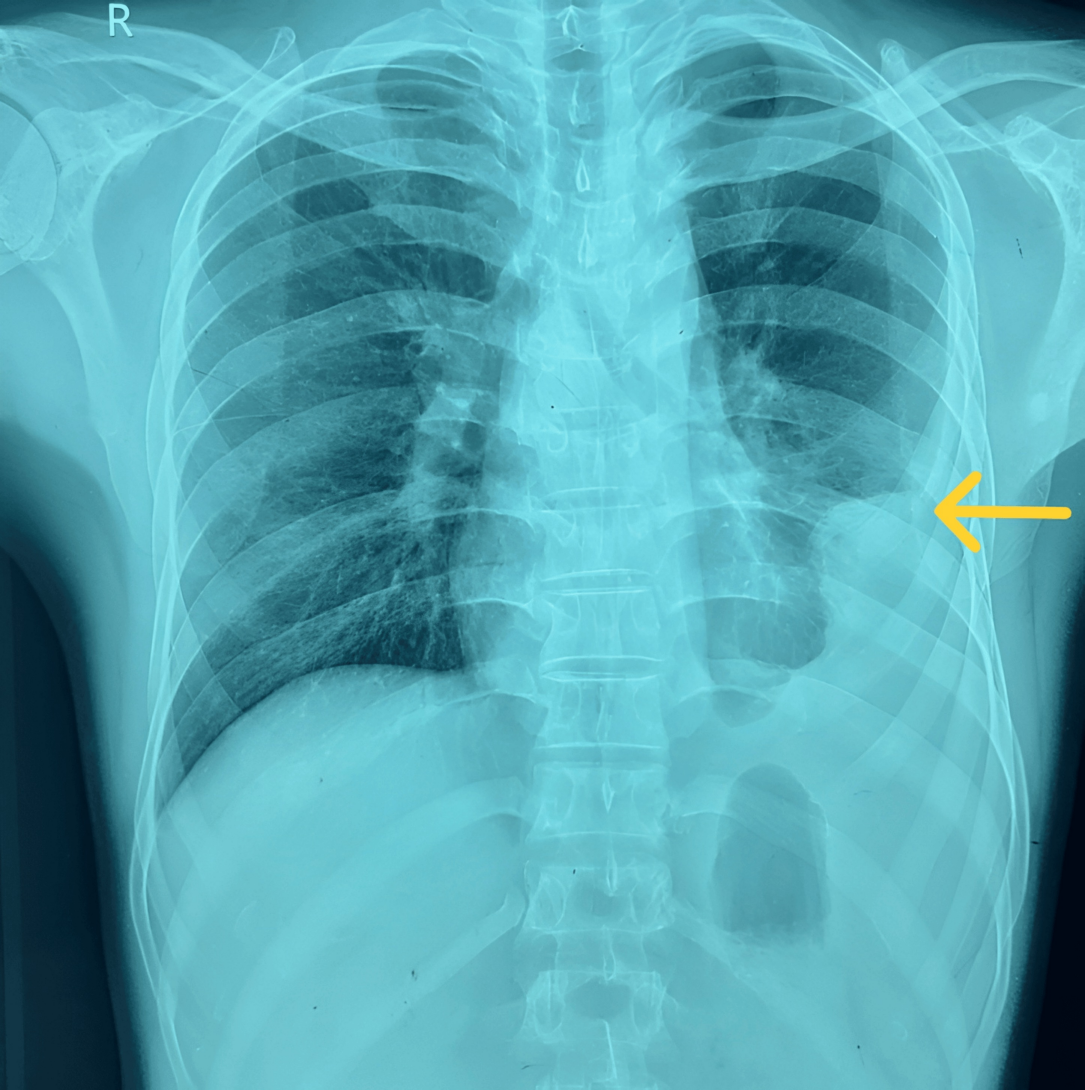
Chest X-ray shows a left-sided pleural effusion (yellow arrow).

Chest radiography showed a left-sided pleural effusion involving the lower and middle zones (Figure 1). Sputum acid-fast bacillus testing and sputum GeneXpert MTB/RIF were negative.

Pleural fluid analysis was consistent with a lymphocyte-predominant exudative effusion (Table 2). Electrocardiography demonstrated sinus rhythm, and a 2D echocardiogram was normal.

**Table 2:** Pleural fluid analysis. Pleural fluid laboratory parameters measured at the time of admission. The markedly elevated protein concentration with lymphocyte-predominant cellularity is consistent with an exudative pleural effusion. Other parameters were within acceptable limits.

Pleural fluid	On admission value	Reference range
Protein	62 g/L	<30 g/L
Glucose	6.4 mmol/L	3.3-5.6 mmol/L
Red cells	80/mm³	<1,000/mm³
White cells	52/mm³ (1 polymorph, 51 lymphocytes)	<1,000/mm³, predominantly mononuclear cells

**Figure 2:**
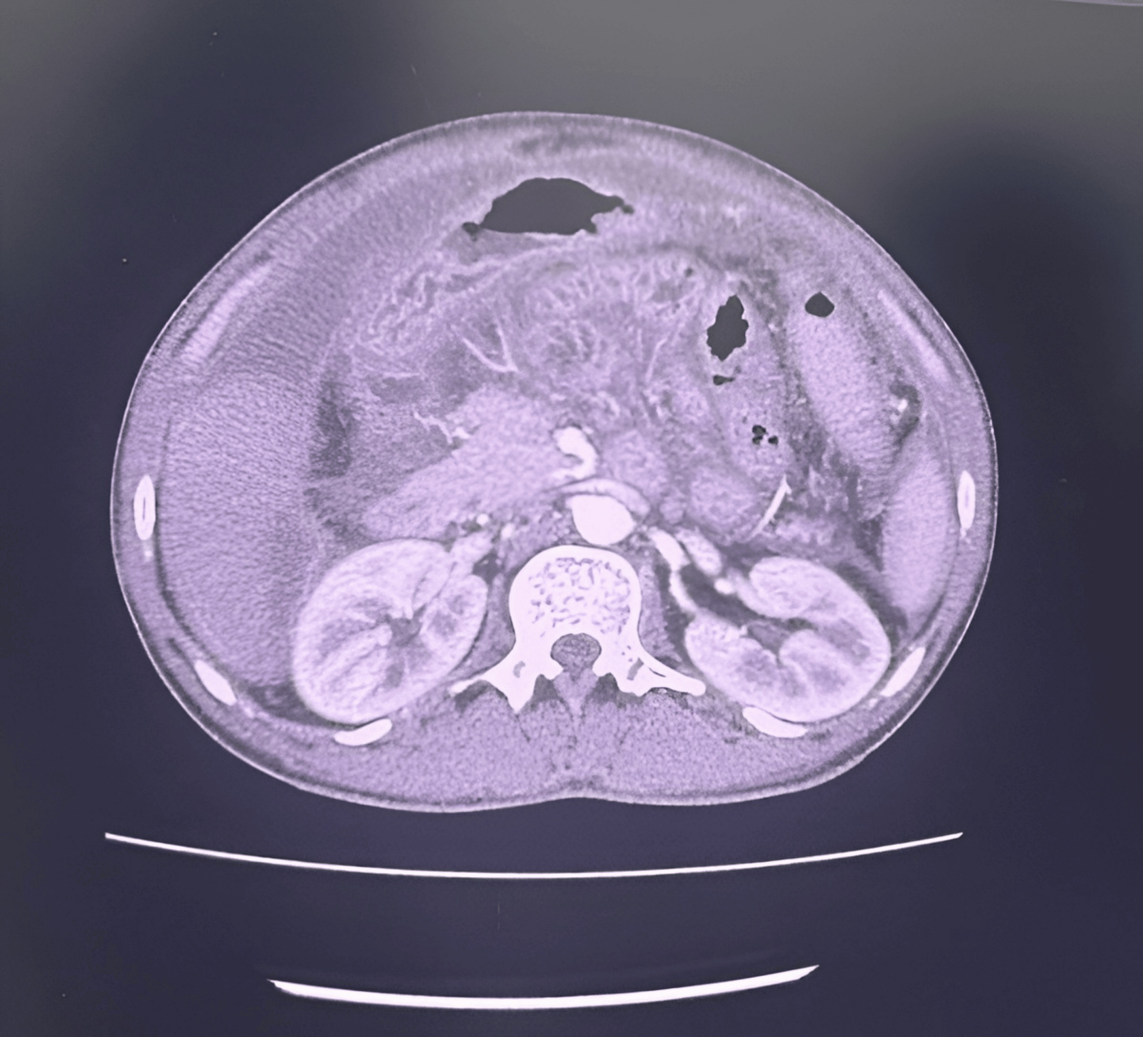
Contrast-enhanced computed tomography of the abdomen demonstrating gross ascites.

**Figure 3:**
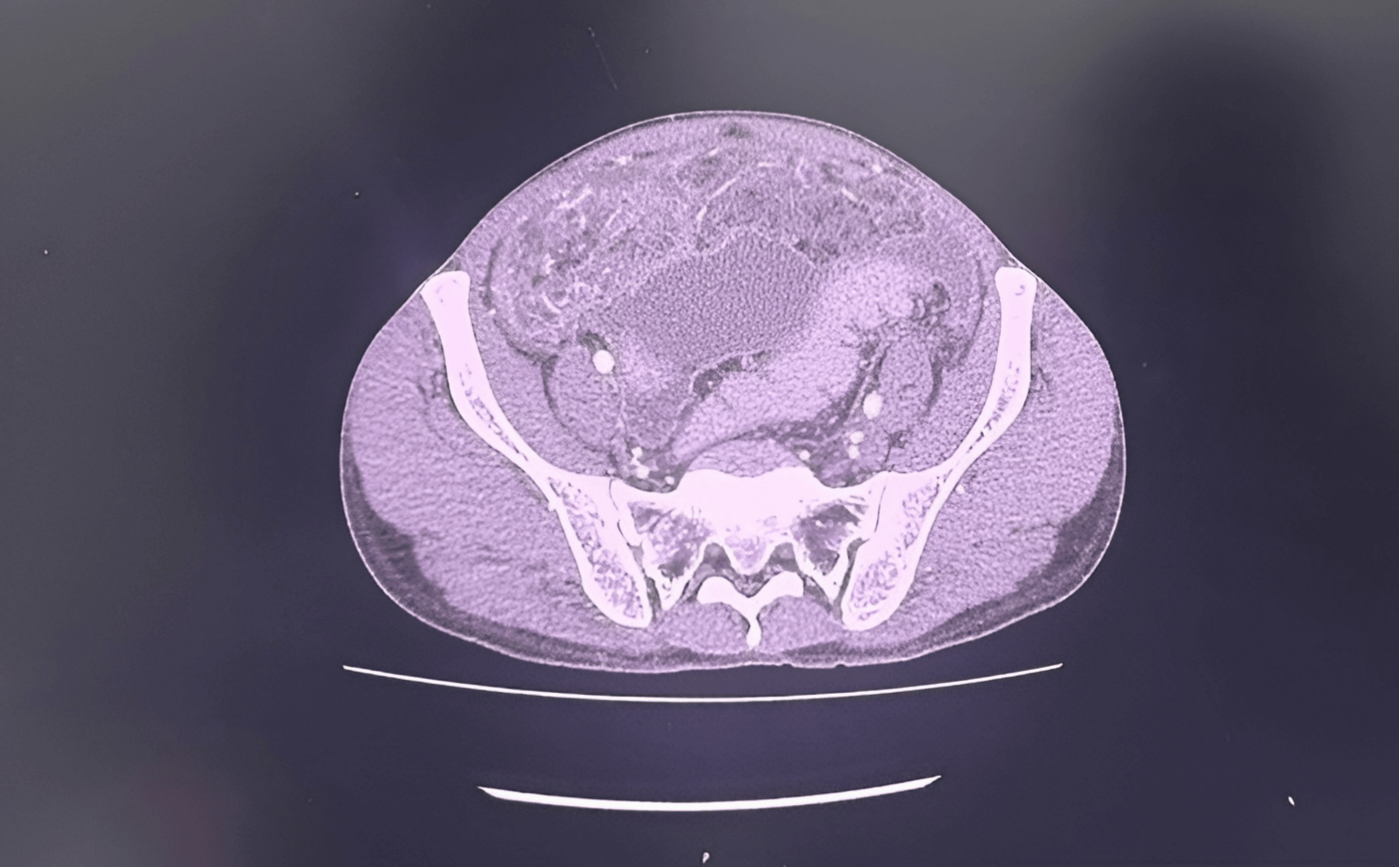
Contrast-enhanced computed tomography of the abdomen demonstrating omental thickening.

Contrast-enhanced CT (CECT) of the abdomen demonstrated omental haziness with gross ascites (Figures 2-3).

An omental biopsy was performed, and histopathology revealed fibroconnective tissue with multiple caseating granulomas, consistent with TB. Based on these findings, a diagnosis of peritoneal TB was made. The patient was referred to the Chest Clinic and commenced on standard anti-TB therapy consisting of isoniazid 300 mg/day, rifampicin 600 mg/day, pyrazinamide 1,500 mg/day, and ethambutol 1,500 mg/day for six months. At one-month follow-up, his general condition had improved significantly, and laboratory parameters showed marked improvement (Table 3).

**Table 3:** Comparison of investigation reports during admission and one-month follow-up. AST: Aspartate aminotransferase; ALT: alanine aminotransferase; LDH: Lactate dehydrogenase; INR: International normalized ratio; APTT: activated partial thromboplastin time.

Investigations	On admission	On discharge	One month after discharge	Reference values
WBC	6,400 × 10³/µL	7,000 × 10³/µL	6,400 × 10³/µL	5,000-11,000 × 10³/µL
Haemoglobin	10.5 g/dL	10.4 g/dL	11 g/dL	12-16 g/dL
Platelets	174,000/µL	230,000/µL	265,000/µL	150,000-450,000/µL
AST	39 U/L	41 U/L	30 U/L	15-37 U/L
ALT	32 U/L	38 U/L	34 U/L	12-78 U/L
CRP	78 mg/L	24 mg/L	20 mg/L	0-5 mg/L
Albumin	21 g/L	20 g/L	27 g/L	34-50 g/L
Serum creatinine	55 µmol/L	45 µmol/L	70 µmol/L	53-88 µmol/L
Total bilirubin	7.2 µmol/L	10 µmol/L	8.4 µmol/L	3.4-17.1 µmol/L
LDH	462 U/L	250 U/L	233 U/L	81-234 U/L
INR	1.24	1.3	1	-
APTT	38 s	36 s	38 s	25-38 s
Serum calcium	2.43 mmol/L	2.23 mmol/L	2.3 mmol/L	2.1-2.5 mmol/L

## Discussion

Pulmonary TB is the most common form; however, extrapulmonary involvement accounts for approximately 15-20% of all cases worldwide [1,2]. Abdominal TB remains a significant diagnostic challenge due to its highly variable clinical presentation and frequent overlap with other intra-abdominal conditions such as malignancies, chronic liver disease, and inflammatory bowel disease [1,3]. The disease burden varies geographically, with higher prevalence observed in endemic areas and among immunocompromised individuals [3].

Understanding the underlying mechanisms of abdominal TB is crucial for accurate diagnosis and effective management. Its pathogenesis can occur through several routes. It may arise through reactivation of latent infection, hematogenous dissemination from a primary pulmonary focus, or lymphatic spread from mesenteric lymph nodes; however, hematogenous spread remains the most common mechanism, often associated with concurrent or antecedent tuberculous pleural effusion. Less commonly, abdominal TB may result from ingestion of infected sputum or from direct contiguous extension of infection from adjacent organs [3,4]. Immunosuppressed individuals, particularly those with HIV infection, are more susceptible to extrapulmonary TB due to impaired cell-mediated immunity. In our patient, despite being immunocompetent, residence in a densely populated, TB-endemic area likely contributed to the development of peritoneal TB.

Diagnosis of abdominal TB is often delayed because of its nonspecific clinical features, including abdominal distension, altered bowel habits, fever, and weight loss. These manifestations frequently mimic other conditions such as peritoneal carcinomatosis and cirrhosis. In this case, the subacute onset of ascites and vague gastrointestinal symptoms led to a broad differential diagnosis, necessitating a systematic and multidisciplinary diagnostic approach [3-8].

Ascitic fluid analysis remains a cornerstone in the evaluation of suspected tuberculous peritonitis. However, its diagnostic yield is often limited due to the paucibacillary nature of the disease. Typical findings include a lymphocyte-predominant exudate with a low serum-ascites albumin gradient (SAAG), as observed in our patient. Among the available biomarkers, adenosine deaminase (ADA) is considered one of the most reliable surrogate indicators of tuberculous peritonitis. A recent meta-analysis demonstrated that ADA levels exceeding 100 U/L have a high specificity for abdominal TB, underscoring its value as a diagnostic adjunct in clinical practice [9]. In our patient, despite negative microbiological and PCR results, we were unable to obtain the ADA value due to its unavailability in our hospital setting.

Abdominopelvic imaging is an important tool in evaluating suspected abdominal TB. Although the findings are not specific, imaging can reveal features that strongly suggest the diagnosis, such as peritoneal thickening or nodularity, ascites, omental caking, and intra-abdominal lymphadenopathy [6,7,8,10]. These findings help increase clinical suspicion and guide the need for further invasive investigations, but imaging alone is rarely sufficient to confirm the diagnosis [10].

When non-invasive investigations are inconclusive, a laparoscopic peritoneal biopsy becomes essential, with reported diagnostic yields exceeding 90% [6,7,8]. In our case, due to extensive adhesions, we were unable to proceed with laparoscopy. A mini laparotomy was performed, which revealed diffusely thickened peritoneum measuring approximately 3 mm. All bowel loops were matted together within the abdominal cavity with areas of loculated fluid. Histopathological examination demonstrated fibroconnective tissue containing multiple caseating granulomas, favouring a diagnosis of TB.

Once the diagnosis is confirmed, standard anti-tuberculous therapy remains the cornerstone of management [3,7]. The conventional six-month regimen-consisting of a two-month intensive phase with Isoniazid, Rifampicin, Pyrazinamide, and Ethambutol, followed by a four-month continuation phase with Isoniazid and a Rifampicin-achieves high cure rates. Our patient showed complete clinical recovery, including resolution of ascites, after the prompt initiation of therapy. This case highlights the importance of early recognition, individualized management, and timely treatment initiation in achieving favourable outcomes in abdominal TB [3].

## Conclusions

This study highlights the diagnostic challenges associated with peritoneal TB and emphasizes the importance of early diagnosis and prompt treatment, even in immunocompetent patients with few or no identifiable risk factors, particularly in endemic regions when presenting with vague or inconclusive clinical findings. Conversely, when definitive confirmation is not feasible but clinical, epidemiological, and laboratory findings strongly suggest peritoneal TB, an empiric trial of anti-TB therapy may be justified. However, conditions with similar clinical presentations, such as malignancy and portal hypertension, should always be carefully excluded.
